# Red blood cell folate and visceral adipose tissue among young and middle-aged American adults

**DOI:** 10.3389/fnut.2025.1512410

**Published:** 2025-07-01

**Authors:** Chaohui Jiang, Jianfen Li, Haiyan Yu, Xingda Luo, Lingfeng Wu, Qilian Long, Bin Zhang

**Affiliations:** ^1^Department of Hematopathology, Jiangmen Central Hospital, Jiangmen, China; ^2^Department of General Practice, Jiangmen Central Hospital, Jiangmen, China; ^3^Department of Cardiology, The First Affiliated Hospital, Sun Yat-sen University, Guangzhou, China; ^4^Department of Cardiovascular Disease and Clinical Experimental Center, Jiangmen Central Hospital, Jiangmen, China

**Keywords:** visceral adipose tissue, folate status, red blood cell folate, cross-sectional study, National Health and Nutrition Examination Survey

## Abstract

**Background:**

The relationship between red blood cell (RBC) folate levels and visceral adipose tissue (VAT) accumulation has not been well-explored. This study investigates the association between RBC folate and VAT in young and middle-aged adults in the United States.

**Methods:**

A cross-sectional analysis was conducted using data from 7,591 individuals aged 20 to 59 years, drawn from the National Health and Nutrition Examination Survey (NHANES) 2011–2018. VAT was measured using dual-energy X-ray absorptiometry (DXA). RBC folate levels were quantified using a multi-step process combining microbiologic assays and liquid chromatography–tandem mass spectrometry (LC–MS/MS). Multivariate linear regression models were applied to assess the relationship between RBC folate and VAT mass.

**Results:**

After adjusting for potential confounders, RBC folate levels were positively associated with VAT mass, with a *β* coefficient (95% CI) of 0.05 (0.03, 0.07). A J-shaped dose–response relationship was observed (nonlinear, *p* < 0.001), with a threshold effect identified at an RBC folate level of 397.18 ng/mL. Below this threshold, the association was negative [*β* (95% CI): −0.10 (−0.20, −0.01)], while above the threshold, the association became positive [*β* (95% CI): 0.06 (0.03, 0.09)].

**Conclusion:**

The association between RBC folate and VAT mass in young and middle-aged American adults follows a J-shaped curve, with a threshold effect observed at an RBC folate level of 397.18 ng/mL. Further research is needed to explore the underlying mechanisms of this association.

## Introduction

Obesity has emerged as a significant public health concern due to its rising global prevalence (1). It is associated with an increased risk of numerous non-communicable diseases, including diabetes, non-alcoholic steatohepatitis, cardiovascular diseases, gout, and various cancers (1–4). Recently, obesity has been redefined as a condition characterized by excess adiposity, with clinical obesity specifically recognized as a chronic, systemic illness where excess body fat impairs the function of organs, tissues, or the entire body, potentially leading to severe complications such as cardiovascular disease and renal failure (5). This nuanced definition highlights the need for comprehensive assessment methods beyond body mass index (BMI), emphasizing direct measures of adiposity and functional health impacts (5). Among obesity-related markers, visceral adipose tissue (VAT) is particularly important, with its assessment through computed tomography (CT) or magnetic resonance imaging (MRI) serving as a reliable indicator of metabolic and cardiovascular health (6, 7). Furthermore, abdominal fat accumulation is independently linked to poor physical fitness and chronic low-grade inflammation, regardless of BMI (8). Given VAT’s central role in metabolic dysfunction, understanding the factors that contribute to its accumulation is essential.

Folate, also referred to as vitamin B9, represents a family of water-soluble compounds collectively known as “reduced folates,” such as tetrahydrofolate (THF) and its derivatives (e.g., 5-methyltetrahydrofolate) (9). These biologically active forms are essential for various physiological functions, including DNA synthesis, cell division, and the prevention of neural tube defects (10). Naturally occurring folates are found in a variety of foods, including leafy green vegetables, legumes, citrus fruits, and fortified cereals (9). Folic acid, the synthetic oxidized form of folate, is commonly used in food fortification and dietary supplements. In the United States, mandatory fortification of enriched cereal grain products with folic acid was authorized in 1996 and fully implemented in 1998 (11, 12). Over time, public health concerns have shifted from insufficient folate intake to potential risks associated with excessive intake (13).

Serum folate primarily reflects recent folate intake, and a low level in serum alone cannot differentiate between a temporary drop due to short-term dietary insufficiency and a more prolonged state of folate deficiency (14, 15). In contrast, red blood cell folate levels provide a more accurate assessment of long-term folate status, typically over a period of up to 4 months, and are commonly used to evaluate the effectiveness of folic acid supplementation and correct deficiencies (16). Research suggests that measuring folate in red blood cells offers the most reliable assessment (17).

Previous studies have demonstrated a correlation between elevated RBC folate levels and obesity (18, 19). However, the relationship between RBC folate and visceral adipose tissue remains unclear. Visceral fat accumulation in young and middle-aged adults is a significant predictor of future cardiovascular health, metabolic syndrome, and overall quality of life in later years, making it an important factor to address (20, 21). This age group is particularly relevant for studying visceral fat accumulation, as early development of visceral adiposity can have long-lasting effects on health, and interventions at this stage could significantly reduce future health risks. Therefore, this study aimed to investigate the association between RBC folate and visceral adipose tissue in young and middle-aged adults in the United States, with a focus on the potential role of folate as a determinant of visceral adiposity, based on prior findings linking RBC folate levels to obesity.

## Methods

### Study population

This cross-sectional study utilized data from four cycles of the National Health and Nutrition Examination Survey (NHANES) conducted between 2011–2012, 2013–2014, 2015–2016, and 2017–2018, managed by the Centers for Disease Control and Prevention (CDC). NHANES provides a comprehensive overview of the health and nutritional status of the non-institutionalized U.S. population. Participants were selected through a stratified, multistage probability sampling method, ensuring a representative sample that reflects the demographic diversity of the American population (22). The survey collects a wide range of health-related information, including demographic data, physical examination results, laboratory test outcomes, and dietary habits. Data collection was conducted by the National Center for Health Statistics (NCHS) following ethical guidelines and with approval from an ethics review board. Informed consent was obtained from all participants prior to their inclusion in the survey. The NHANES dataset is publicly available on the official website.^1^ For this study, only individuals aged 20 years and older who completed the survey were included. Pregnant women and participants with incomplete data on dual-energy X-ray absorptiometry (DXA) scans, RBC folate, or covariates were excluded.

### Assessment of RBC folate

In the NHANES 2011–2018, RBC folate was assessed using a multi-step process. Initially, whole blood folate concentration was measured using a microbiologic assay. This measurement was then adjusted for RBC volume. Subsequently, serum total folate concentration, calculated as the sum of individual serum folate forms, was used to correct the RBC folate values. Individual serum folate forms were quantified using liquid chromatography–tandem mass spectrometry (LC–MS/MS). For folate forms with concentrations below the limit of detection (LOD), values were imputed as LOD divided by the square root of 2. The serum folate forms included in the calculation of total folate were 5-methyl-tetrahydrofolate, folic acid, 5-formyl-tetrahydrofolate, tetrahydrofolate, and 5,10-methenyl-tetrahydrofolate. This comprehensive approach, combining microbiologic assays with LC–MS/MS measurements, ensured a thorough evaluation of RBC folate levels.

### Assessment of VAT mass

Whole-body DXA scans were performed on NHANES participants aged 8–59 years using Hologic Discovery model A densitometers. DXA is widely used for body composition analysis due to its high precision and efficiency. In this study, we specifically analyzed VAT mass at the L4-L5 vertebral level within the abdominal cavity, as measured using Hologic APEX 4.0 software. To meet our inclusion criteria, we included only participants aged 20 to 59 years who completed the survey. VAT mass was reported in grams.

### Covariates

A comprehensive set of covariates, derived from existing literature, was evaluated to account for potential confounders (23–25). These covariates included demographic, socioeconomic, lifestyle, and health-related factors, such as age, sex, marital status, race/ethnicity, education level, poverty income ratio (PIR), body mass index (BMI), height, smoking status, alcohol consumption, physical activity, energy intake, total carbohydrates, total protein intake, total fat intake, estimated glomerular filtration rate (eGFR), and albumin levels. Race/ethnicity was classified into the following categories: non-Hispanic White, non-Hispanic Black, Mexican American, and other racial/ethnic groups (26). Marital status was grouped into two categories: individuals who were married or living with a partner, and those living alone (27). Education levels were stratified into three groups: less than high school, high school or equivalent, and more than high school (26). Family income was divided into three categories based on the PIR. PIR was categorized as ≤1.30, 1.31–3.50, and >3.50 (28). Smoking status was classified into three groups: never smokers (having smoked fewer than 100 cigarettes in their lifetime), current smokers, and former smokers (those who quit after smoking more than 100 cigarettes) (27). Alcohol consumption was self-reported and categorized as never (fewer than 12 drinks in a lifetime), former (having consumed ≥12 drinks in 1 year but not in the past year), mild (women consuming ≤1 drink and men ≤2 drinks per day), moderate (women consuming ≤2 drinks and men ≤3 drinks per day), or heavy (women consuming ≥3 drinks and men ≥4 drinks per day) (28). Physical activity was quantified based on the metabolic equivalent of task (MET) criteria, expressed in minutes per week (29). Renal function was assessed by calculating the eGFR using the Chronic Kidney Disease Epidemiology Collaboration (CKD-EPI) equation, a validated method for evaluating kidney function.

Dietary intake was assessed using two non-consecutive 24-h dietary recalls, with the average intake calculated for the analysis. The variables analyzed included total energy intake, total carbohydrates, total protein intake, and total fat intake. Folate intake was not included as a covariate due to collinearity with red blood cell folate levels, though specific folate intake data are provided in Table 1. Total folate intake, expressed as Dietary Folate Equivalents (DFE), accounts for the differing bioavailability of folate from natural food sources and synthetic folic acid (1 μg food folate = 1 μg DFE; 1 μg synthetic folic acid with food = 1.7 μg DFE) (30). Since folic acid is mandatorily added to grain products in the United States, total starch intake was calculated by subtracting total sugar and total dietary fiber from total carbohydrate intake to examine the relationship between starch consumption and RBC folate levels. This variable represents the combined intake of folate from natural sources and folic acid from fortified foods. The nutrient composition of recalled food items was determined using the USDA Food and Nutrient Database for Dietary Studies (FNDDS).^2^

**Table 1:** Baseline characteristics by red blood cell folate level.

Characteristics	Total	Red blood cell folate, ng/mL			*P*-value
	*n* = 7,591	T1(<386)	T2(386–525)	T3(>525)	
		*n* = 2,528	*n* = 2,495	*n* = 2,568	
Age, Mean (SD), years	39.35 (11.47)	37.52 (11.56)	38.90 (11.40)	41.57 (11.08)	< 0.001
Sex, n (%)					0.001
Male	3,628 (47.79)	1,247 (49.33)	1,228 (49.22)	1,153 (44.9)	
Female	3,963 (52.21)	1,281 (50.67)	1,267 (50.78)	1,415 (55.1)	
Race/ethnicity, n (%) ^a^					< 0.001
Non-Hispanic White	2,936 (38.68)	710 (28.09)	967 (38.76)	1,259 (49.03)	
Non-Hispanic Black	1,651 (21.75)	826 (32.67)	470 (18.84)	355 (13.82)	
Mexican American	1,026 (13.52)	323 (12.78)	378 (15.15)	325 (12.66)	
Other Hispanic	715 (9.42)	248 (9.81)	256 (10.26)	211 (8.22)	
Others ^b^	1,263 (16.64)	421 (16.65)	424 (16.99)	418 (16.28)	
Marital status, n (%)					< 0.001
Married or living with a partner	4,551 (59.95)	1,362 (53.88)	1,494 (59.88)	1,695 (66)	
Living alone	3,040 (40.05)	1,166 (46.12)	1,001 (40.12)	873 (34)	
PIR, n (%)					< 0.001
≤1.3	2,425 (31.95)	929 (36.75)	783 (31.38)	713 (27.76)	
>1.3–3.5	2,710 (35.70)	888 (35.13)	910 (36.47)	912 (35.51)	
>3.5	2,456 (32.35)	711 (28.12)	802 (32.14)	943 (36.72)	
Education level, n (%)					< 0.001
Less than high school	1,193 (15.72)	454 (17.96)	415 (16.63)	324 (12.62)	
High school or equivalent	1,614 (21.26)	578 (22.86)	528 (21.16)	508 (19.78)	
Above high school	4,784 (63.02)	1,496 (59.18)	1,552 (62.2)	1736 (67.6)	
BMI, Mean (SD), kg/m^2^	29.25 (7.11)	28.48 (7.13)	29.11 (6.91)	30.14 (7.19)	< 0.001
Height, Mean (SD), cm	168.15 (9.85)	168.24 (9.68)	168.11 (9.91)	168.08 (9.98)	0.821
Waist-to-hip ratio, Mean (SD)	2.93 (0.26)	2.90 (0.26)	2.92 (0.25)	2.97 (0.26)	< 0.001
Waist-to-height ratio, Mean (SD)	0.58 (0.10)	0.57 (0.10)	0.58 (0.10)	0.60 (0.10)	< 0.001
Smoking status, n (%)					< 0.001
Never	4,600 (60.60)	1,502 (59.41)	1,529 (61.28)	1,569 (61.1)	
Former	1,330 (17.52)	345 (13.65)	435 (17.43)	550 (21.42)	
Now	1,661 (21.88)	681 (26.94)	531 (21.28)	449 (17.48)	
Alcohol consumption, n (%)					0.002
Never	967 (12.74)	347 (13.73)	301 (12.06)	319 (12.42)	
Former	741 (9.76)	221 (8.74)	223 (8.94)	297 (11.57)	
Mild	2,517 (33.16)	826 (32.67)	814 (32.63)	877 (34.15)	
Moderate	1,483 (19.54)	489 (19.34)	511 (20.48)	483 (18.81)	
Heavy	1883 (24.81)	645 (25.51)	646 (25.89)	592 (23.05)	
Physical activity MET, mean (SD), min/week	4227.41 (6387.36)	4568.08 (6816.34)	4327.23 (6378.56)	3795.07 (5920.52)	< 0.001
Energy intake, Mean (SD), kcal/day	2143.56 (851.25)	2094.94 (859.90)	2164.39 (841.50)	2171.19 (850.38)	0.002
Total carbohydrate intake, Mean (SD), g/day	256.47 (109.37)	252.37 (112.99)	257.51 (107.27)	259.51 (107.68)	0.056
Total starch intake, Mean (SD), g/day	128.74 (58.54)	124.73 (59.29)	130.37 (57.50)	131.11 (58.61)	< 0.001
Total protein intake, Mean (SD), g/day	84.50 (37.33)	81.81 (37.98)	86.31 (36.82)	85.39 (37.04)	< 0.001
Total fat intake, Mean (SD), g/day	82.08 (39.12)	79.29 (38.45)	83.34 (39.24)	83.59 (39.53)	< 0.001
Total folate intake, Mean (SD), (μg DFE/day)	534.20 (331.39)	478.11 (301.57)	541.05 (327.40)	582.77 (354.30)	< 0.001
Serum total folate, Mean (SD), ng/ mL	17.11 (11.64)	11.57 (5.20)	16.34 (6.42)	23.34 (16.26)	< 0.001
eGFR, Mean (SD), mL/ (min·1.73 m^2^)	102.82 (18.03)	103.90 (17.81)	103.48 (17.65)	101.12 (18.50)	< 0.001
Albumin, Mean (SD), g/dL	4.29 (0.34)	4.30 (0.34)	4.30 (0.34)	4.28 (0.34)	0.053
Subcutaneous fat mass, Mean (SD), g	1682.07 (853.89)	1587.15 (886.72)	1664.50 (834.76)	1792.58 (826.70)	< 0.001
Total lean mass, Mean (SD), g	51821.63 (12761.21)	51003.77 (12363.35)	51932.86 (12667.25)	52527.09 (13198.37)	< 0.001
VAT mass, Mean (SD), g	501.81 (277.75)	450.33 (255.40)	494.34 (272.85)	559.73 (292.48)	< 0.001
Cycle year, n (%)					< 0.001
2011–2012	2074 (27.32)	835 (33.03)	669 (26.81)	570 (22.2)	
2013–2014	2,414 (31.80)	705 (27.89)	803 (32.18)	906 (35.28)	
2015–2016	2077 (27.36)	670 (26.5)	653 (26.17)	754 (29.36)	
2017–2018	1,026 (13.52)	318 (12.58)	370 (14.83)	338 (13.16)	

T, tertiles; SD, standard deviation; PIR, poverty income ratio; BMI, body mass index; MET, metabolic equivalent of task; DFE, Dietary Folate Equivalents; VAT, visceral adipose tissue; eGFR, estimated glomerular filtration rate. ^a^Race and ethnicity were self-reported. ^b^Includes multiracial participants. NHANES does not provide a detailed list of all races and ethnicities. *P* < 0.05 was set as the threshold of statistical significance.

### Statistical analysis

Unweighted counts and percentages are reported for categorical data, while continuous variables are presented as means with corresponding standard deviations. One-way analysis of variance (ANOVA) was used to assess differences in continuous variables across groups, and chi-square tests were employed to evaluate differences in categorical variables. To examine the relationship between RBC folate and VAT mass, a multivariate linear regression model was applied. Results are presented as regression coefficients (*β*) with 95% confidence intervals (CI). In Model 1, adjustments were made for demographic variables, including age, sex, marital status, race/ethnicity, PIR, and education level. Model 2 included additional adjustments for lifestyle factors, such as smoking status, alcohol consumption, physical activity, energy intake, total fat intake, total carbohydrate intake, and total protein intake, in addition to the variables adjusted for in Model 1. Model 3 incorporated further adjustments for height, eGFR, and albumin levels, in addition to the variables included in Model 2. Finally, Model 4 included additional adjustments for BMI, eGFR, and albumin levels, alongside the variables adjusted for Model 2.

A restricted cubic spline regression model was used to examine the dose–response relationship between RBC folate levels and visceral adipose tissue VAT mass. The knots for the spline were placed at the 5th, 35th, 65th, and 95th percentiles of RBC folate. This model was adjusted for the covariates specified in Model 4. Additionally, a two-piecewise linear regression model was applied to identify potential threshold effects, adjusting for relevant confounders.

Linear regression was used for interaction and subgroup analyses, stratified by age, sex, race/ethnicity, PIR, smoking status, alcohol consumption, physical activity, and BMI, with stratification factors adjusted for a spectrum of covariates in Model 4. Age was categorized into two groups: 20–39 years and 40–59 years. This division ensures balanced subgroup sizes, and the study by Shen et al. suggests that 40 years may be a key transition point in aging, with significant physiological and metabolic changes occurring around this age, particularly in cardiovascular health, lipid metabolism, and immune regulation (31). Physical activity was classified into three levels: low (MET score <600 per week), moderate (600–3,000), and vigorous (>3,000) (32). BMI was categorized into three groups: <25, 25–29.9, and ≥30 kg/m^2^ (33).

A sensitivity analysis was performed to assess the robustness of the results. Missing covariate data were handled using multiple imputations, which were based on five replications and the chained equation approach implemented in the R mice procedure. This method was employed to maximize statistical power and minimize bias arising from missing data. The covariates imputed included age, sex, PIR, race/ethnicity, education level, marital status, smoking status, alcohol consumption, physical activity, energy intake, total fat intake, total carbohydrate intake, total protein intake, BMI, eGFR, and albumin. Five datasets were generated and analyzed together to produce pooled results.

No formal power calculation was performed, as the sample size was determined by the available data. All analyses were conducted using R Statistical Software (Version 4.2.2,^3^ The R Foundation) and the Free Statistics analysis platform (Version 1.9.2, Beijing, China).^4^ Descriptive statistics were computed for all participants, and statistical significance was evaluated using two-tailed tests with a *p*-value of <0.05.

## Results

### Baseline characteristics of the study population

**Figure 1:**
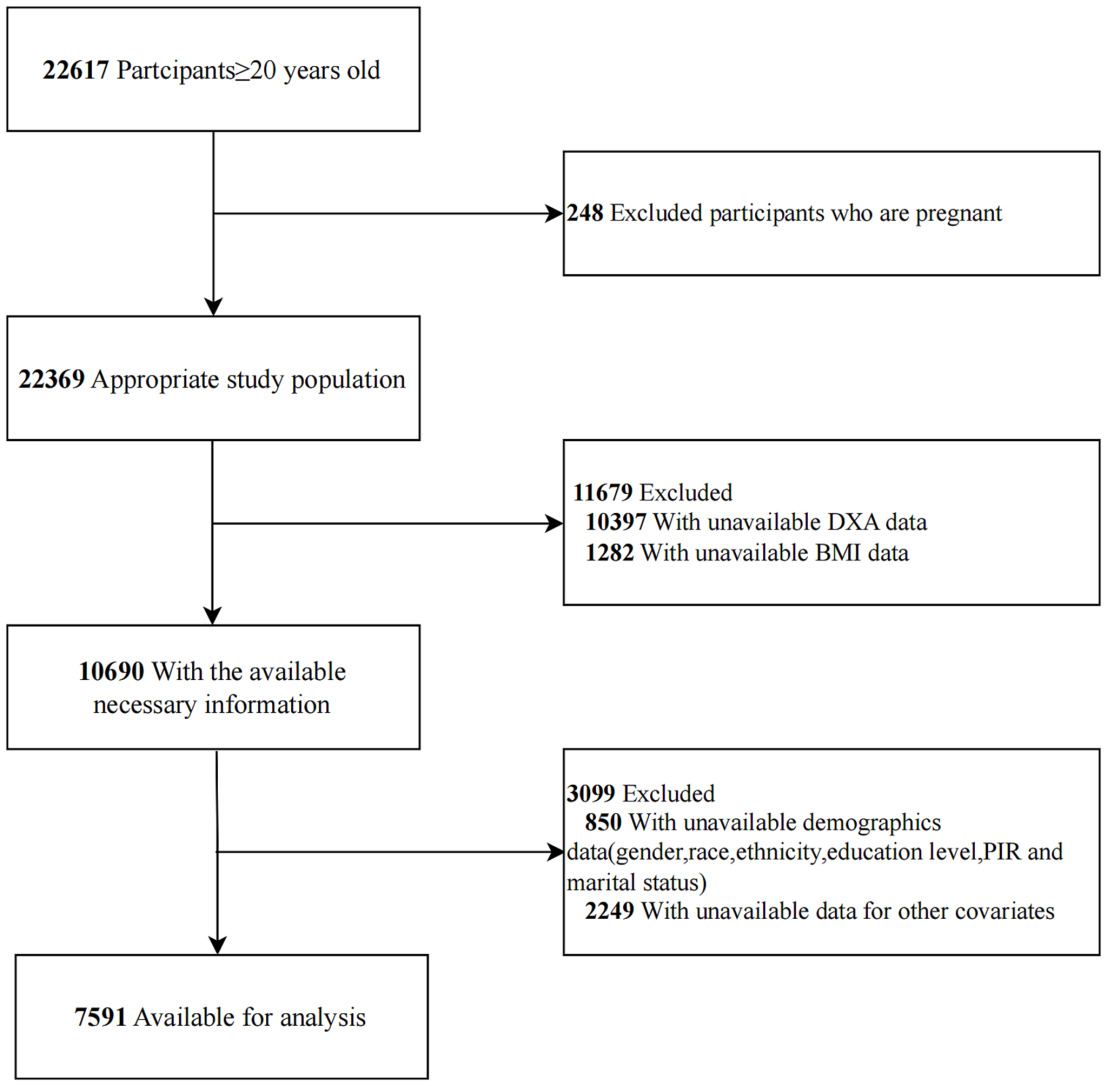
Flowchart of participant selection from NHANES 2011–2018.

A total of 7,591 individuals met the inclusion criteria and were included in the final analysis. The selection process is detailed in Figure 1. The baseline characteristics of participants aged 20–59 years are presented in Table 1. The mean age of participants was 39.35 (11.47) years, with 3,628 (47.79%) being male. Participants were stratified into three groups based on tertiles of RBC folate: Tertile 1 (T1, *n* = 2,528; <386 ng/mL), Tertile 2 (T2, *n* = 2,495; 386–525 ng/mL), and Tertile 3 (T3, *n* = 2,568; >525 ng/mL). The mean visceral adipose tissue (VAT) mass was 501.81 (277.75) g, with T1 exhibiting lower VAT levels compared to the other groups.

Elevated RBC folate levels were observed among older adults, females, non-Hispanic White individuals, who were married or cohabiting, nonsmokers, and those with lower levels of physical activity. These higher RBC folate levels were also associated with increased BMI, higher waist - to - hip ratio, higher waist - to - height ratio, greater subcutaneous fat mass, and higher total lean mass. Additionally, elevated RBC folate levels were linked to higher educational attainment, as well as increased energy intake, greater total carbohydrate intake, higher starch intake, and elevated total folate and fat intake. Lower eGFR and higher serum folate levels were also found to be associated with elevated RBC folate levels.

### Relationship between RBC folate and VAT

Univariate analysis revealed significant associations between VAT mass and several factors, including age, sex, race/ethnicity, marital status, education level, smoking status, alcohol consumption, physical activity, BMI, height, total fat intake, total carbohydrate intake, starch intake, and total folate intake. Additionally, VAT mass was found to be associated with albumin levels, eGFR, and RBC folate levels (Table 2).

**Table 2:** Association of covariates and visceral adipose tissue mass.

Covariates	*β*(95%CI)	*P*-value
Age, years	10.14 (9.65,10.64)	< 0.001
Sex		
Female (versus male)	−45.85 (−58.32, −33.38)	< 0.001
Race/ethnicity (versus non-Hispanic White)		
Non-Hispanic Black	−103.93 (−120.28, −87.58)	< 0.001
Mexican American	62.63 (43.35,81.9)	< 0.001
Other Hispanic	−14.09 (−36.25,8.07)	0.213
Others	−106.31 (−124.19, −88.43)	< 0.001
Education level (versus less than high school)		
High school or equivalent	−23.65 (−44.32, −2.98)	0.025
Above high school	−74.24 (−91.76, −56.73)	< 0.001
Marital status (versus married or living with a partner)		
Living alone	−68.6 (−81.26, −55.94)	< 0.001
PIR (versus ≤1.3)		
>1.3–3.5	−6.45 (−21.66,8.75)	0.405
>3.5	−31.5 (−47.07, −15.93)	< 0.001
Smoking status (versus never)		
Former	95.93 (79.12,112.74)	< 0.001
Now	36.48 (21.02,51.93)	< 0.001
Alcohol consumption (versus never)		
Former	97.03 (70.63,123.43)	< 0.001
Mild	−15.77 (−36.22,4.69)	0.131
Moderate	−22.68 (−45.03, −0.34)	0.047
Heavy	3.88 (−17.52,25.27)	0.723
Physical activity MET, min/week	0 (0,0)	< 0.001
BMI, kg/m2	25.7 (25.03,26.36)	< 0.001
Height, cm	1.57 (0.93,2.2)	< 0.001
Energy intake, kcal/day	0 (−0.01,0.01)	0.763
Total carbohydrate intake, g/day	−0.07 (−0.12, −0.01)	0.026
Total starch intake, g/day	−0.18 (−0.29, −0.07)	< 0.001
Total protein intake, g/day	−0.04 (−0.21,0.13)	0.651
Total fat intake, g/day	0.18 (0.02,0.34)	0.028
Total folate intake, μg DFE/day	−0.05 (−0.06, −0.03)	< 0.001
Serum total folate, ng/ mL	−0.49 (−1.03,0.05)	0.073
eGFR, mL/ (min·1.73 m^2^)	−2.4 (−2.75, −2.06)	< 0.001
Albumin, g/dL	−165.85 (−183.92, −147.78)	< 0.001
Red blood cell folate, ng/mL	0.28 (0.25,0.31)	< 0.001

PIR, poverty income ratio; MET, metabolic equivalent of task; DFE, Dietary Folate Equivalents; eGFR, estimated glomerular filtration rate; BMI, body mass index; CI, confidence interval. The unit for continuous variables and the reference group for categorical variables are provided next to the variables. *P* < 0.05 was set as the threshold of statistical significance.

The results of the multivariable linear regression analysis examining the association between RBC folate and VAT mass are summarized in Table 3. After adjusting for potential confounders in Model 3, RBC folate was positively associated with VAT mass [*β* (95% CI), 0.15 (0.13, 0.18)]. Participants in the second tertile (T2: 386–525 ng/mL) and third tertile (T3: >525 ng/mL) had an increase in VAT mass of 15.88 g (95% CI, 2.77–28.99) and 52.34 g (95% CI, 38.91–65.77), respectively, compared to those in the lowest tertile (T1: <386 ng/mL). After further adjustment for potential confounders in Model 4, RBC folate remained positively associated with VAT mass [*β* (95% CI), 0.05 (0.03, 0.07)]. Participants in the third tertile (T3: >525 ng/mL) exhibited an increase in VAT mass of 15.04 g (95% CI, 5.21–24.87) compared to those in the lowest tertile (T1: <386 ng/mL). The *p* for trend was 0.003.

**Table 3:** Association between red blood cell folate and visceral adipose tissue mass.

Red blood cell folate, ng/mL	Crude		Model 1		Model 2		Model 3		Model 4	
	*β*(95%CI)	*P*-value	*β*(95%CI)	*P*-value	*β*(95%CI)	*P*-value	*β*(95%CI)	*P*-value	*β*(95%CI)	*P*-value
Continuous	0.28 (0.25,0.31)	<0.001	0.17 (0.14,0.19)	<0.001	0.16 (0.13,0.19)	<0.001	0.15 (0.13,0.18)	<0.001	0.05 (0.03,0.07)	<0.001
Tertiles										
T1(<386)	0(Reference)		0(Reference)		0(Reference)		0(Reference)		0(Reference)	
T2(386–525)	44.01 (28.85,59.17)	<0.001	18.41 (4.9,31.92)	0.008	18.16 (4.7,31.62)	0.008	15.88 (2.77,28.99)	0.018	−0.34 (−9.9,9.23)	0.945
T3(>525)	109.4 (94.34,124.45)	<0.001	57.64 (43.86,71.42)	<0.001	56.56 (42.78,70.34)	<0.001	52.34 (38.91,65.77)	<0.001	15.04 (5.21,24.87)	0.003
*P* for trend		<0.001		<0.001		<0.001		<0.001		0.003

Model 1: adjusted for age, sex, poverty income ratio, race/ethnicity, education level, and marital status. Model 2: adjusted for model 1 + smoking status, alcohol consumption, physical activity, energy intake, total fat intake, total carbohydrate intake, and total protein intake. Model 3: adjusted for model2 + height, estimated glomerular filtration rate, and albumin. Model 4: adjusted for model2 + body mass index, estimated glomerular filtration rate, and albumin. T, tertiles; CI, confidence interval. *P* < 0.05 was set as the threshold of statistical significance.

**Figure 2:**
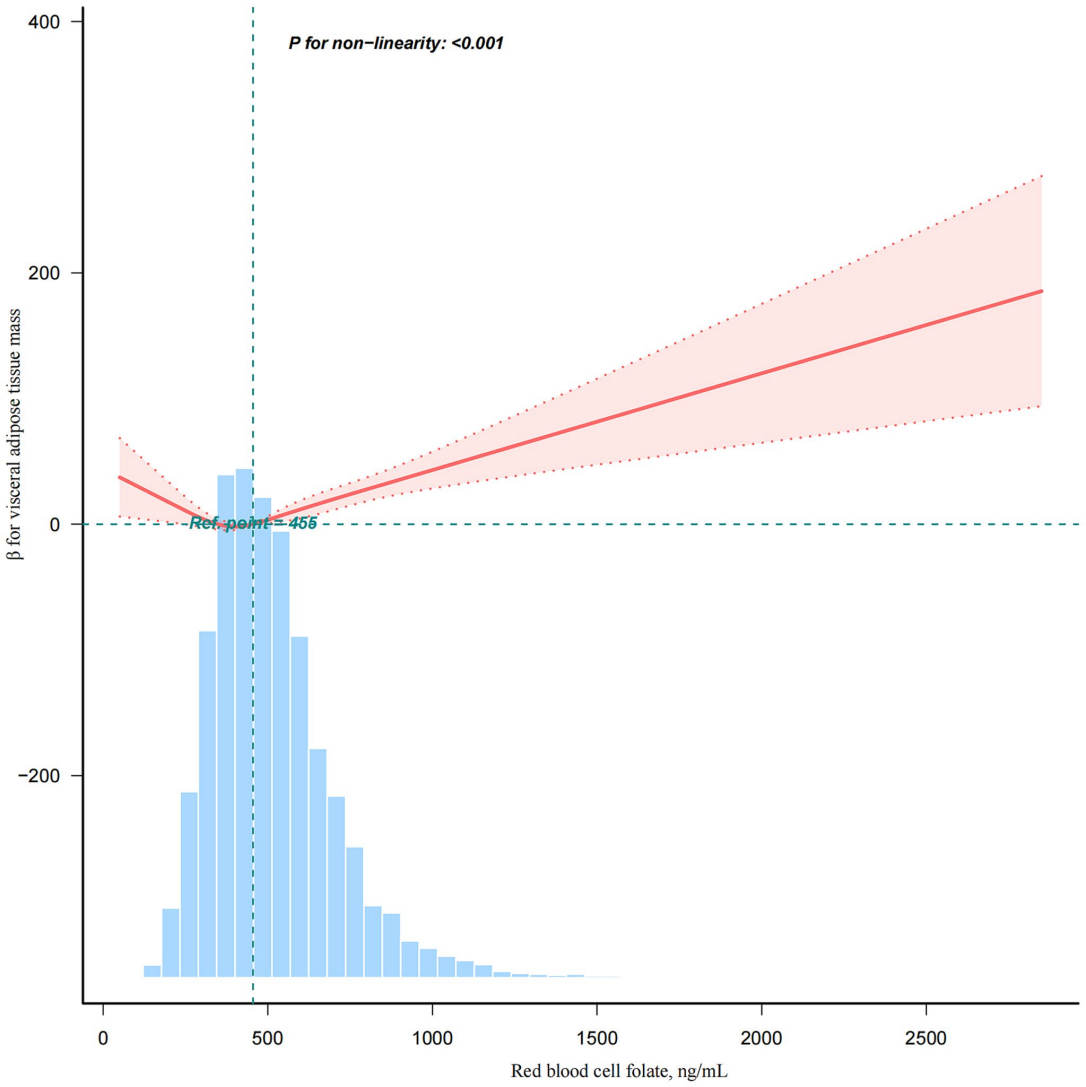
A non-linear association between red blood cell folate levels and visceral adipose tissue mass. This analysis was adjusted for age, sex, poverty income ratio, race/ethnicity, education level, marital status, smoking status, alcohol consumption, physical activity, energy intake, total fat intake, total carbohydrate intake, total protein intake, body mass index, estimated glomerular filtration rate, and albumin.

The dose–response relationship between RBC folate and VAT mass followed a nonlinear J-shaped curve (Figure 2; *p* for non-linearity < 0.001). A threshold effect was identified at an RBC folate level of 397.18 ng/mL (Table 4). Below this threshold, the association was negative [*β* (95% CI): −0.10 (−0.20, −0.01)], while above the threshold, the association became positive [*β* (95% CI): 0.06 (0.03, 0.09)].

**Table 4:** Threshold effect analysis of the relationship of red blood cell folate with visceral adipose tissue mass.

Red blood cell folate, ng/mL	Adjusted model ^a^	
	*β*(95%CI)	*P*-value
< 397.18	−0.10(−0.20, −0,01)	0.035
≥397.18	0.06 (0.03, 0.09)	<0.001
Likelihood Ratio test		<0.001

^a^Adjusted for age, sex, poverty income ratio, race/ethnicity, education level, marital status, smoking status, alcohol consumption, physical activity, energy intake, total fat intake, total carbohydrate intake, total protein intake, body mass index, estimated glomerular filtration rate, and albumin. CI, confidence interval. *P* < 0.05 was set as the threshold of statistical significance.

### Subgroup analyses

**Figure 3:**
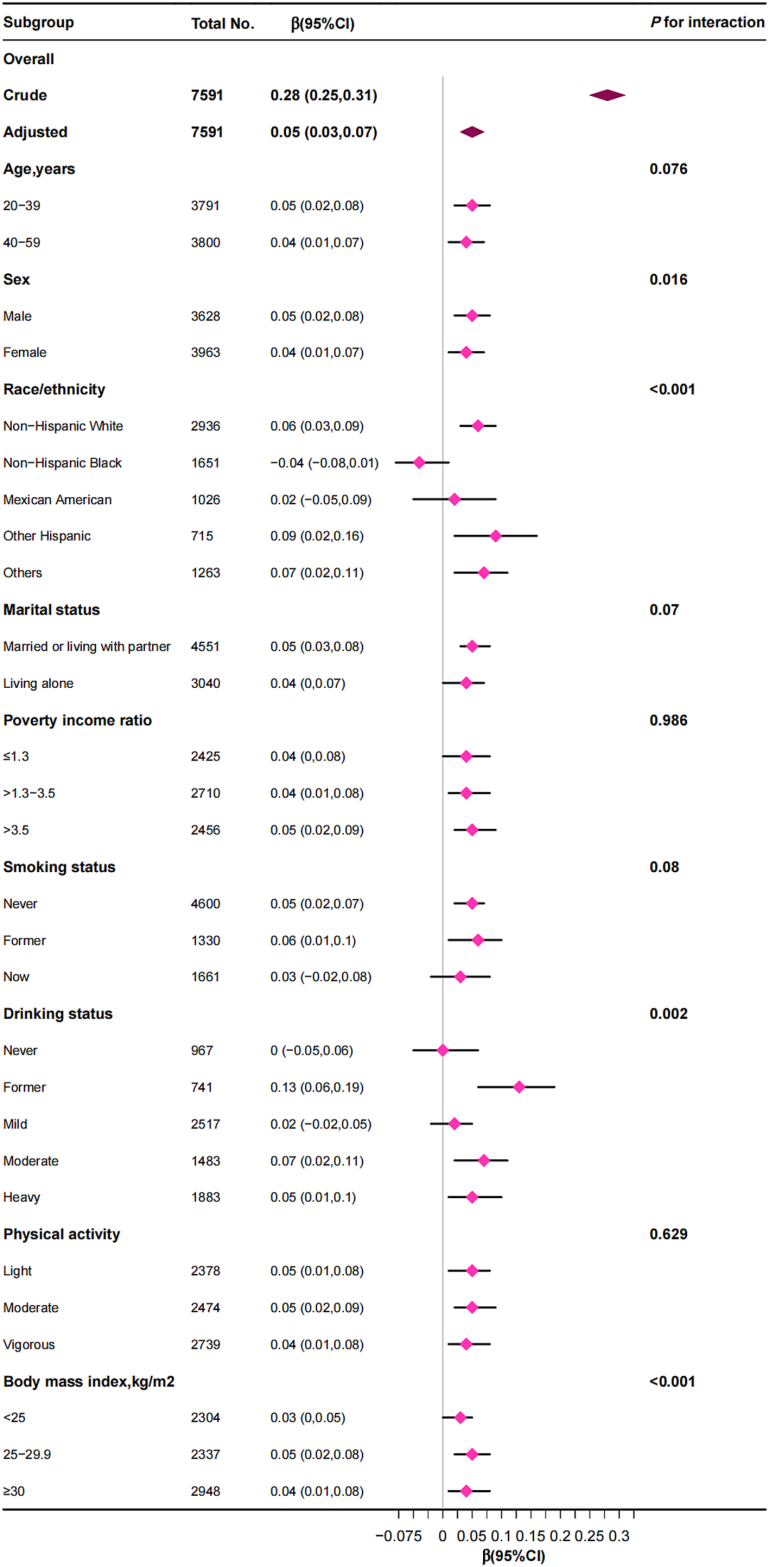
The correlation between red blood cell folate levels and visceral adipose tissue mass was examined, with stratification factors adjusted for a spectrum of covariates encompassing age, sex, poverty income ratio, race/ethnicity, education level, marital status, smoking status, alcohol consumption, physical activity, energy intake, total fat intake, total carbohydrate intake, total protein intake, body mass index, estimated glomerular filtration rate, and albumin.

Subgroup analyses indicated that the association between RBC folate and VAT was generally consistent across various demographic and lifestyle factors, including age, sex, marital status, PIR, smoking status, alcohol consumption, physical activity, and BMI (Figure 3). However, significant interactions were observed for sex, race/ethnicity, alcohol consumption, and BMI. While RBC folate was positively correlated with VAT in most subgroups, a distinct finding emerged among non-Hispanic Black individuals, where RBC folate was negatively associated with VAT [*β* (95% CI), −0.04 (−0.08 to 0.01)]. Additionally, the positive correlation between RBC folate and VAT persisted across all BMI categories (<25, 25–29.9, and ≥30 kg/m^2^).

### Sensitivity analyses

To address missing covariate data for 10,690 participants, we conducted multiple imputations with five iterations. Five datasets were created and analyzed together to produce pooled results (Table 5). After adjusting for confounders in Model 4, RBC folate remained positively associated with VAT mass [*β* (95% CI), 0.05 (0.03, 0.07)]. Participants in the third tertile (T3: >525 ng/mL) exhibited an increase in VAT mass of 15.27 g (95% CI, 5.41–25.12) compared to those in the lowest tertile (T1: <386 ng/mL).

**Table 5:** Sensitivity analysis: association between red blood cell folate and visceral adipose tissue mass using pooled results from five imputed datasets.

Red blood cell folate, ng/mL	Total no.^a^	Crude		Adjusted^b^	
		*β*(95%CI)	*P*-value	*β*(95%CI)	*P*-value
Continuous	10,690	0.27 (0.24, 0.30)	<0.001	0.05 (0.03, 0.07)	<0.001
Tertiles					
T1(<386)	3,669	0(Reference)		0(Reference)	
T2(386–525)	3,566	43.48 (30.98, 55.99)	<0.001	−0.20 (−9.78, 9.37)	0.967
T3(>525)	3,455	105.31 (92.71, 117.92)	<0.001	15.27 (5.41, 25.12)	0.002

^a^Multiple imputations of missing data (five datasets). ^b^Adjusted for age, sex, poverty income ratio, race/ethnicity, education level, marital status, smoking status, alcohol consumption, physical activity, energy intake, total fat intake, total carbohydrate intake, total protein intake, body mass index, estimated glomerular filtration rate, and albumin. T, tertiles; CI, confidence interval. *P* < 0.05 was set as the threshold for statistical significance.

## Discussion

This cross-sectional study identified a threshold effect at an RBC folate level of 397.18 ng/mL, indicating a non-linear relationship between RBC folate levels and VAT mass. Below this threshold, the association was negative, while above it, the association became positive.

According to WHO guidelines (34), RBC folate levels above 400 ng/mL (906 nmol/L) are typically considered sufficient for preventing neural tube defect-affected pregnancies in women of reproductive age. However, this threshold is specific to that population and does not apply to the general population. Our study suggests that RBC folate may influence visceral fat distribution, particularly when levels exceed 397.18 ng/mL. However, there is no consensus on a general RBC folate threshold for the population, and further research is needed to establish a broader reference range. Previous studies show that both low and high RBC folate levels are linked to metabolic disturbances and obesity (18, 35, 36), which aligns with the threshold effect we observed. This highlights the complexity of folate’s role in metabolic health and suggests that the impact of folate on metabolic outcomes may vary across different ranges.

While the relationship between RBC folate and VAT has been underexplored, our findings support previous studies indicating a link between RBC folate and metabolic markers such as BMI and metabolic diseases (17, 18, 33, 36). For example, Bird et al. found that while obesity is associated with lower serum folate levels and dietary intake, RBC folate is positively correlated with obesity, suggesting that RBC folate may be a more reliable marker of folate status in obese individuals (18). Li et al. demonstrated that elevated RBC folate levels were associated with an increased risk of NAFLD, a condition linked to central obesity (33). Our study further supports RBC folate as a potential biomarker for metabolic dysfunction (33).

In our analysis, we adjusted for height in Model 3 and for BMI in Model 4 to examine these associations. Although the effect size decreased significantly with the inclusion of BMI, the association remained statistically significant. All subsequent analyses, including curve fitting, threshold analysis, and subgroup analysis, also accounted for BMI. Our findings align with previous research suggesting a link between RBC folate levels and obesity. Specifically, we found that when BMI was included as a covariate, the effect size decreased, indicating that RBC folate levels are associated with obesity in a broader sense, not limited to visceral fat alone. This suggests that both visceral and non-visceral fat mass may be linked to RBC folate levels.

However, after adjusting for BMI in Model 4 and in subsequent analyses, the association between RBC folate and VAT remained statistically significant. We also observed consistent positive correlations between RBC folate and VAT across all BMI subgroups (<25, 25–29.9, and ≥30 kg/m^2^). The results suggest that the relationship between RBC folate and VAT may exist independently of BMI. These findings underscore the importance of conducting further cohort studies to better understand the causal relationship between RBC folate and VAT, particularly in the context of obesity and metabolic dysfunction.

In subgroup analyses, interaction effects were also observed with sex, race, and alcohol consumption. First, in the sex subgroup, the effect size for women was *β*  = 0.04, while for men it was *β*  = 0.05. This difference is likely due to estrogen’s protective effect on visceral fat accumulation in women, particularly in young and middle-aged adults (37). Second, subgroup analyses indicated a potential negative association between RBC folate and VAT among non-Hispanic Black individuals. This finding may reflect genetic predispositions, dietary patterns, or variations in folate metabolism unique to this population. Racial and ethnic differences in obesity and fat distribution are well-documented and may partially explain these findings (38, 39). Furthermore, the methylenetetrahydrofolate reductase (MTHFR) 677C → T polymorphism, a genetic variant that reduces enzyme activity and alters folate metabolism, is more prevalent in Asian and Latin American populations but less common in African populations (40). Third, interaction effects were observed within the alcohol consumption subgroup, likely because alcohol intake is related to both folate metabolism and obesity (9, 41).

In the United States, folate is primarily added to cereals through a mandatory fortification program, which has led to increased folate intake, particularly from carbohydrate-rich fortified foods, such as breakfast cereals, commonly consumed by many individuals. As shown in Table 1, carbohydrate and starch intake are positively correlated with RBC folate levels. However, the univariate analysis in Table 2 reveals a negative correlation between carbohydrate and starch intake and VAT, which may seem counterintuitive. Carbohydrates typically come from both healthy sources (e.g., whole grains) and less healthy sources (e.g., refined grains and processed foods). Previous studies have shown that excessive carbohydrate intake, particularly from refined sources, is associated with obesity and metabolic disturbances. The “carbohydrate-insulin model” has been proposed to explain how excessive carbohydrate consumption can lead to obesity and its downstream cardiometabolic complications (42, 43). However, some studies suggest that the consumption of high-quality carbohydrates, such as whole grains (particularly oats and barley) and legumes, is associated with weight loss, as well as a reduction in the risk of diabetes, cardiovascular disease, and cardiovascular mortality (44). Additionally, resistant starch (RS), the indigestible portion of starch, has been studied for its potential effects on reducing obesity (45). One possible explanation for the negative correlation between carbohydrate intake and VAT in our study is that increased grain consumption, particularly through fortified foods, may be linked to reduced fat intake, which could result in less pronounced VAT accumulation. This relationship, however, remains complex, and further research is needed to fully understand how carbohydrate intake, especially from fortified foods, influences fat distribution and metabolic outcomes.

The relationship between RBC folate levels and visceral adipose tissue (VAT) accumulation is complex and not fully understood. One potential explanation is that obesity itself alters folate metabolism, potentially leading to higher RBC folate levels. In obese individuals, impaired nutrient absorption and altered fat storage dynamics can contribute to changes in folate status (46–49). Additionally, vitamin B12 plays a critical role in lipid metabolism, and its deficiency can disrupt lipid synthesis, insulin resistance, and mitochondrial function—all of which are associated with obesity-related metabolic disturbances (50, 51). Elevated methylmalonic acid (MMA), a biomarker of B12 deficiency, is often observed in these conditions. Studies suggest that high folate levels in the presence of B12 deficiency may exacerbate metabolic disturbances (52, 53), further complicating the relationship between folate status and VAT accumulation. However, whether elevated RBC folate levels directly contribute to VAT accumulation or merely reflect broader metabolic changes related to obesity remains unclear. Further research is needed to clarify these mechanisms and their implications for metabolic health.

Although the NHANES database employs weighted analysis to make the data representative of the U. S. population, our study did not apply weights. The primary focus of our analysis was the association between RBC folate and VTA mass, rather than estimating the prevalence of visceral obesity in the general U. S. population. As such, we used unweighted data to ensure the analysis accurately reflects the characteristics of our study sample, without adjustments intended to generalize to the broader population. This approach minimizes potential biases from weighting procedures and enhances the transparency of our data representation.

This study has several strengths, including its use of the NHANES database, which is a nationally representative and demographically diverse dataset of U. S. adults. It is the first to establish a dose–response relationship between RBC folate and VAT, and the comprehensive adjustment for multiple covariates enhances the validity of the findings. Additionally, the application of multiple imputations for sensitivity analysis helps mitigate potential bias from missing data. However, there are limitations. The study sample was restricted to U. S. adults aged 20–59 years, limiting the generalizability of the results to other age groups or populations. Moreover, the cross-sectional design precludes causal inferences. Longitudinal studies are needed to elucidate the causal mechanisms linking RBC folate to VAT accumulation.

## Conclusion

In conclusion, this study identifies a non-linear relationship between RBC folate levels and VAT, with a threshold effect at an RBC folate level of 397.18 ng/mL. These findings underscore the need for further research to elucidate the mechanisms underlying this relationship, particularly in the context of obesity and metabolic disturbances. While RBC folate holds potential as a biomarker for metabolic risk, longitudinal studies are essential to establish causality and better understand how changes in folate status may influence VAT accumulation.

## Data Availability

Publicly available datasets were analyzed in this study. This data can be found here: The data supporting this study’s findings are available in NHANES at: https://www.cdc.gov/nchs/nhanes/index.htm.

## Footnotes

^1^ http://www.cdc.gov/nchs/nhanes.htm

^2^ http://www.ars.usda.gov/ba/bhnrc/fsrg

^3^ http://www.R-project.org

^4^ http://www.clinicalscientists.cn/freestatistics
